# Early Prognostic Utility of Gp210 Antibody-Positive Rate in Primary Biliary Cholangitis: A Meta-Analysis

**DOI:** 10.1155/2019/9121207

**Published:** 2019-10-13

**Authors:** Chunyang Huang, Weijia Han, Chuanmin Wang, Yanmin Liu, Yue Chen, Zhongping Duan

**Affiliations:** ^1^Difficult & Complicated Liver Diseases and Artificial Liver Center, Beijing Youan Hospital, Capital Medical University, China; ^2^Department of Immunologic Liver Disease, Beijing Youan Hospital, Capital Medical University, Beijing, China; ^3^Beijing Municipal Key Laboratory of Liver Failure and Artificial Liver Treatment Research, Beijing, China; ^4^Department of Infectious Diseases, Taihe Hospital, Shiyan, Hubei, China

## Abstract

**Background:**

The prevalence of primary biliary cholangitis (PBC), which is an autoimmune liver disease, has increased over time. PBC often leads to severe consequences, such as liver failure and death. Stratification tools using biochemical liver tests are needed to assess and predict the progression of this disease at the time of PBC diagnosis.

**Methods:**

We searched PubMed, Cochrane Library, Web of Science, and Embase for studies focused on the relationship between positive rates of Gp210 antibodies and poor prognosis of PBC. The primary end point was the number of PBC patients with poor outcome in the Gp210 antibody (+) and Gp210 antibody (−) groups. The secondary end point was the basic serum level of alanine aminotransferase (ALT), alkaline phosphatase (ALP), total bilirubin (TBIL), and IgM in the two groups. The age and number of female patients were also measured.

**Results:**

A total of 5 studies, comprising 737 patients, were included in this analysis. A positive rate of Gp210 antibodies was positively correlated with poor outcomes and with many types of progression in PBC, especially liver failure. Mortality was also higher in the Gp210 antibody (+) group. Furthermore, the serum levels of ALP and IgM were associated with the positive rate of Gp210 antibodies, while the serum levels of ALT and TBIL were not. The age and number of female patients were also not associated with the positive rate of Gp210 antibodies.

**Conclusion:**

PBC-specific Gp120 antibodies are optimal predictors of PBC prognosis at the time of diagnosis. Some other liver function indicators, such as ALP and IgM, can be used as predictors to complement Gp210 antibodies to establish a stratification tool to predict the prognosis of PBC at the time of diagnosis.

## 1. Introduction

Primary biliary cholangitis (PBC) is an autoimmune disease with an incidence of 0.9 to 5.8 per 100 000 people worldwide. The prevalence of PBC has increased over time due to increased environmental triggers. PBC often leads to liver failure, cirrhosis, and even death. So, it is important to predict the progression of PBC. Although liver biopsy is the gold standard to assess the severity of PBC, it is often limited by pain, invasiveness, interobserver disparity, and sampling error. Stratification tools, using biochemical liver tests applied after 1 year of ursodeoxycholic acid (UDCA) exposure, can readily identify patients with or without sufficient treatment response. For example, global score [1] and UK score [2] are useful for predicting PBC prognosis and the therapeutic effect of UDCA. However, these tools lack early predictive ability and cannot predict PBC prognosis at the time of diagnosis. So, a noninvasive, simple, and reliable method is needed to better assess and predict PBC progression at the time of diagnosis [3, 4].

Gp210 antibodies are highly specific for PBC. This type of antibody, with an integral glycoprotein of the nuclear pore complex, is typical of antinuclear antibodies. Some detection methods, such as a dual isotype ELISA, have been designed to provide enhanced detection of Gp210 antibodies [5]. A meta-analysis has shown that Gp210 antibody positivity is an important diagnostic marker for PBC [6].

Some researchers have described the association between Gp210 antibodies and severe PBC prognosis. They have shown that Gp210 antibody (+) at the time of diagnosis is a strong risk factor for progression to end-stage hepatic failure and have described the clinical significance of Gp210 antibodies in monitoring PBC [7]. However, large samples and multicenter studies are needed to confirm the correlation between Gp210 antibody (+) rate and prognosis of PBC [8].

In this study, we summarize the currently published literature that has analyzed the relationship between Gp210 antibody (+) rate and prognosis of PBC. We aimed to evaluate the value of Gp210 antibodies in predicting poor prognosis of PBC at the time of PBC diagnosis. We also aimed to evaluate whether other liver function indicators at the time of PBC diagnosis can be used as predictors to complement Gp210 antibodies in predicting poor PBC prognosis. We hope to provide new ideas for further PBC management.

## 2. Material and Methods

We followed the methods of a published article by Yao et al. [9]. The processes of study retrieval and analysis were as follows.

### 2.1. Study Selection

This meta-analysis was conducted and reported according to the Preferred Reporting Items for Systematic Reviews and Meta-Analyses (PRISMA) [10]. We searched for articles from January 1990 to April 2019 using the databases of Cochrane Library, Web of Science, Embase, and PubMed. We selected all articles about prognosis of patients with PBC. The following search terms were used: (Primary biliary cirrhosis OR Liver Cirrhoses Biliary OR Biliary Cirrhosis OR Cirrhosis Biliary OR Secondary Biliary Cholangitis OR Biliary Cholangitis Secondary OR Cholangitis Secondary Biliary OR Secondary Biliary Cholangitides OR Secondary Biliary Cirrhosis OR Cirrhosis, Secondary Biliary OR Biliary Cirrhosis Secondary OR Liver Cirrhosis, Obstructive OR Obstructive Liver Cirrhosis OR Primary Biliary Cholangitis OR Biliary Cholangitis Primary OR Cholangitis Primary Biliary OR Primary Biliary Cholangitides OR Biliary Cirrhosis Primary 1 OR Biliary Cirrhosis Primary OR Cholangitis Chronic Nonsuppurative Destructive OR Primary Biliary Cirrhosis) AND (prognosis OR Prognoses OR Prognostic Factors OR Factor Prognostic OR Factors Prognostic OR Prognostic Factor) AND (gp 210 OR gp210).

Two investigators (C.H. and W.H.) conducted a preliminary search separately, sifted through relevant headings and abstracts, deleted duplicate records, and identified relevant terms for further evaluation. References to retrieved articles were also reviewed to identify other eligible studies.

The Ethics Committee of Beijing Youan Hospital approved the study protocol.

### 2.2. Definition and Study End Points

PBC was diagnosed by increased antimitochondrial antibodies (AMAs) in a patient with increased alkaline phosphatase (ALP), assuming other intrahepatic and extrahepatic causes of cholestasis have been excluded [3]. This study contained two end points: (1) number of PBC patients with poor outcome in the Gp210 antibody (+) group and the Gp210 antibody (−) group. Adverse outcomes were defined as occurrence of PBC-related complications including ascites, variceal hemorrhage, hepatic encephalopathy, and high levels of total bilirubin (TBIL) [11], and (2) the serum levels of alanine aminotransferase (ALT), ALP, TBIL, and IgM in the Gp210 antibody (+) and Gp210 antibody (−) groups, and age and number of female patients in the two groups.

### 2.3. Data Extraction and Quality Assessment

Two investigators (C.H. and W.H.) extracted the following information independently from the selected studies: first author; year of publication; age and sex; number of patients enrolled in the Gp210 antibody (+) and Gp210 antibody (−) groups; number of patients with poor prognosis, including adverse vital signs, liver failure, and death in the two groups; and the liver function indicators, including ALT, ALP, TBIL, and IgM in the two groups. When research on the same patients appeared in multiple articles, to avoid duplication of information, we selected the study with the largest sample.

The Newcastle-Ottawa scale (NOS) quality assessment was used to evaluate bias risks in each study.

### 2.4. Study Eligibility

Inclusion criteria were as follows [12, 13]: (1) chronic cholestasis after exclusion of other causes of liver disease, (2) unexplained elevation of serum ALP, (3) positivity of AMA, and (4) liver biopsy which was used to substantiate the diagnosis, but was rarely needed.

Exclusion criteria were as follows: positive serological test for hepatitis B or C virus and comorbidity of primary sclerosing cholangitis, alcoholic liver disease, hemochromatosis, Wilson's disease, a1-antitrypsin deficiency, and presence of complications of cirrhosis.

### 2.5. Statistical Analysis

We used Review Manager 5.2 and Stata 12.0 software for statistical analysis. Differences were expressed as relative risk (RR) with 95% confidence interval (CI) or standard mean difference (SMD) with 95% CI. Heterogeneity was tested using the *I*^2^ statistic. Heterogeneity was considered to be low in studies with *I*^2^ 25–50%, moderate in studies with *I*^2^ 50–75%, and high in studies with *I*^2^ > 75%. *I*^2^ > 50% represented significant heterogeneity. A fixed-effects model was used when study heterogeneity was not significant, and a random-effects model when heterogeneity was significant. Begg's test was used to estimate publication bias, and sensitivity analysis to test stability.

## 3. Results

### 3.1. Study Selection and Characteristics

**Figure 1:**
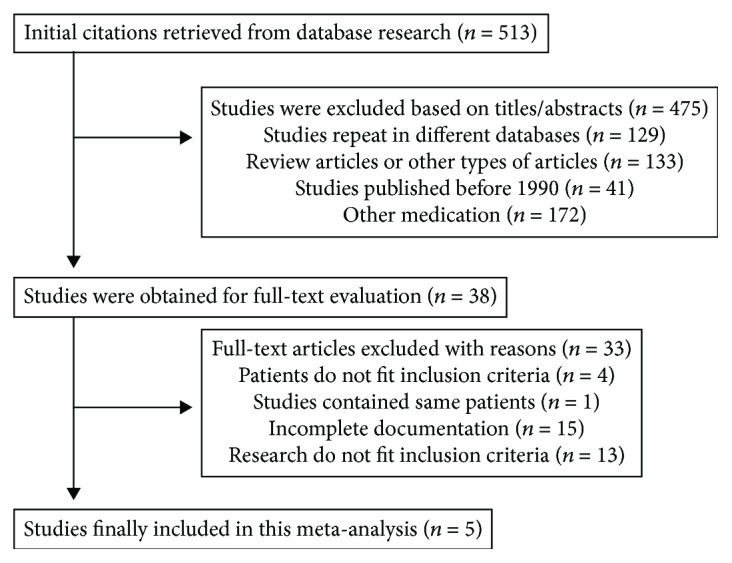
Flow diagram of study selection.

The selection process is illustrated in Figure 1. A total of 5 articles met the inclusion criteria [11, 14–17]. The main characteristics of the included studies are described in Table 1. The meta-analysis included 737 patients from East Asia, comprising 77 male and 660 female patients. There were 239 patients in the Gp210 antibody (+) group and 498 in the Gp210 antibody (−) group. Four of the 5 studies compared the incidence of poor prognosis between the two groups [11, 14–16]. Three of the 5 studies compared death toll between the groups [11, 14, 15]. Three of the 5 studies compared the serum levels of ALT, ALP, TBIL, and IgM between the groups [14, 15, 17].

### 3.2. Quality Assessment

All the studies were retrospective. The results of NOS quality assessment are shown in Table 2. The definition of case and control was all adequate, representative, and comparable. All the studies used the same method of exposure in the case and control groups. Ascertainment of exposure was conducted unblindedly.

### 3.3. Incidence of Poor Prognosis in the Gp210 Antibody (+) and Gp210 Antibody (−) Groups

**Figure 2:**
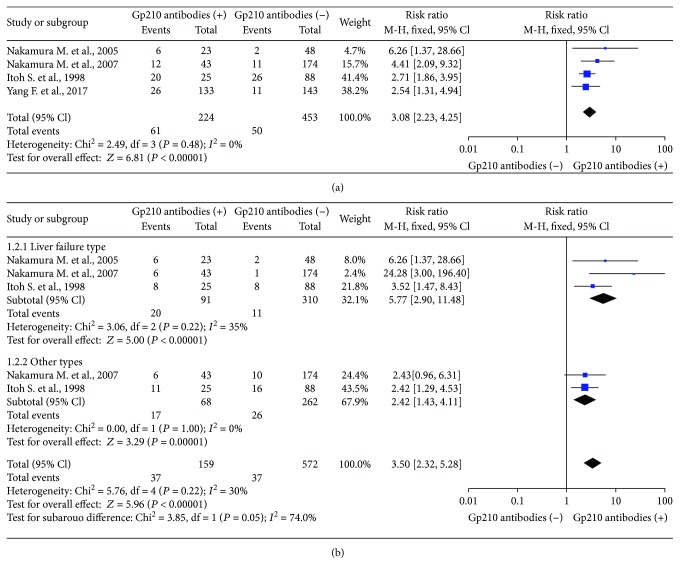
Meta-analysis of relationship between Gp210 antibody-positive rate and poor prognosis. (a) Comparisons of the incidence of poor prognosis between the Gp210 antibody (+) group and the Gp210 antibody (-) group. (b) Subgroup analysis of the incidence of different types of PBC progression.

**Figure 3:**
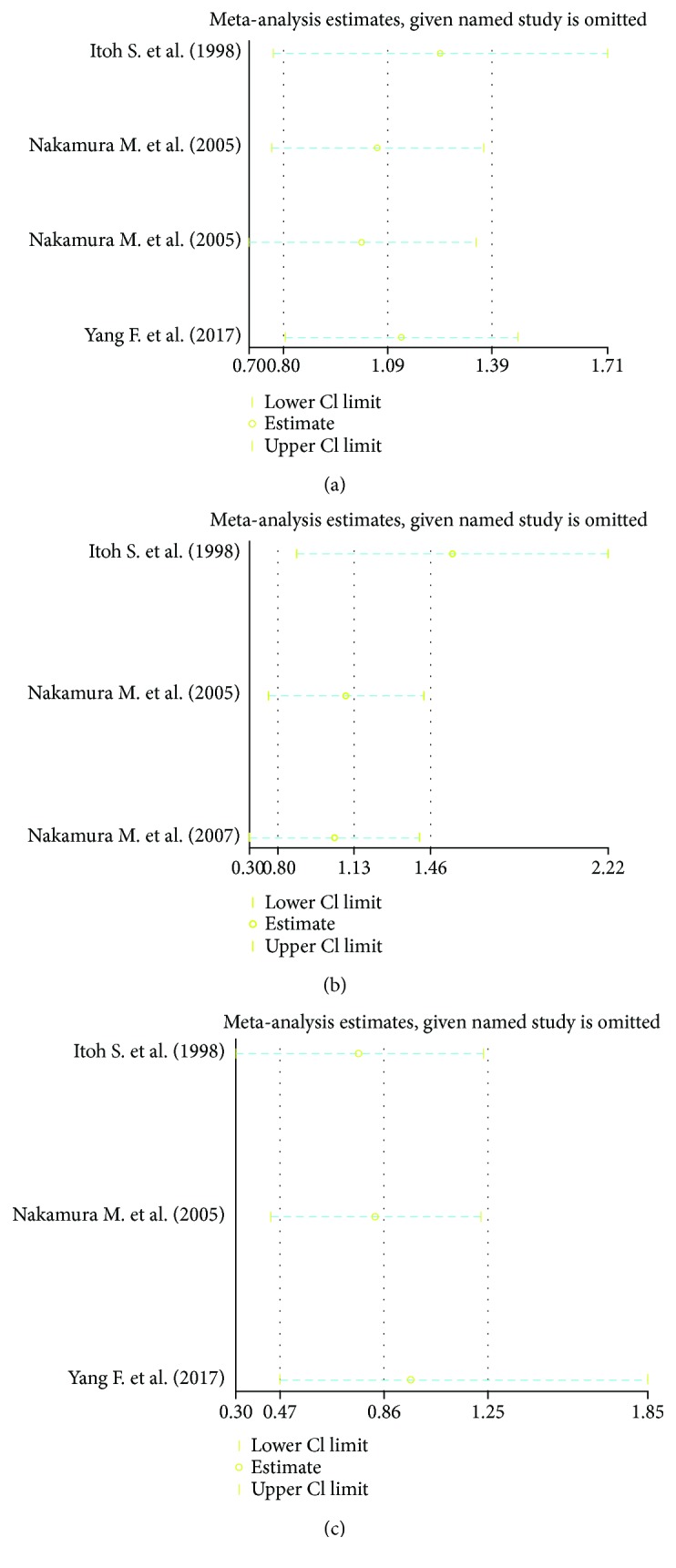
Sensitivity test for forest analysis. (a) Sensitivity analysis for Figure 2(a); (b) Sensitivity analysis for Figure 2(b); (c) Sensitivity analysis for Figure 5.

**Figure 4:**
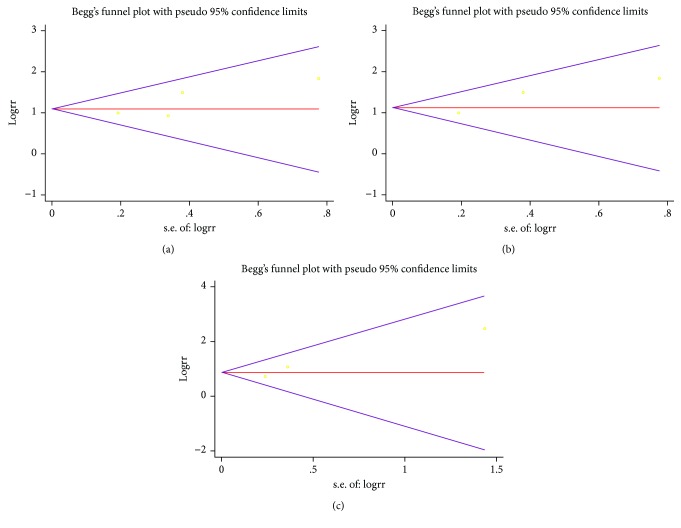
Begg's test for forest analysis. (a) Begg's test for Figure 2(a); (b) Begg's test for Figure 2(b); (c) Begg's test for Figure 5.

We selected 4 studies that measured the incidence of poor prognosis in the Gp210 antibody (+) and Gp210 antibody (−) groups [11, 14–16]. The incidence of poor prognosis was higher in the Gp210 antibody (+) group (RR = 3.08, 95% CI: 2.23–4.25). There was no heterogeneity (*I*^2^ = 6%) (Figure 2(a)). Analysis of sensitivity confirmed the stability of this result (1.09, 95% CI: 0.8–1.39), and the 95% CI for all articles was 0.7–1.71 (Figure 3(a)). Begg's test showed publication bias in these 4 studies, although it was not significant (Pr > ∣*z*∣ = 0.308, continuity corrected) (Figure 4(a)).

Three of these 4 studies measured the incidence of different types of progression, such as liver failure, between the Gp210 antibody (+) and Gp210 antibody (−) groups [11, 14, 16]. We selected these 3 studies to make a subgroup analysis. The incidence of liver failure was higher in the Gp210 antibody (+) group (RR = 5.77, 95% CI: 2.9–11.48). The incidence of other types of progression was also higher in the Gp210 antibody (+) group (RR = 2.42, 95% CI: 1.43–4.11). The differences between the two groups were significant (*P* = 0.05) (Figure 2(b)). Analysis of sensitivity confirmed the stability of this result (1.13, 95% CI: 0.80–1.46), and the range for all articles was 0.68–2.22 (Figure 3(b)). Begg's test showed publication bias in these 3 studies, although it was not significant (Pr > ∣*z*∣ = 1, continuity corrected) (Figure 4(b)).

### 3.4. Incidence of Mortality in the Gp210 Antibody (+) and Gp210 Antibody (−) Groups

**Figure 5:**
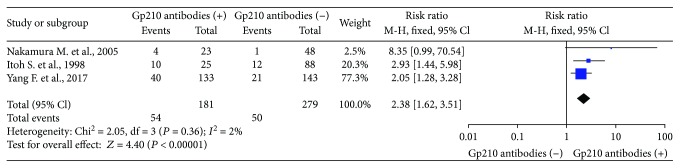
Meta-analysis of relationship between Gp210 antibody-positive rate and incidence of mortality.

We selected 3 studies that measured mortality in the Gp210 antibody (+) and Gp210 antibody (−) groups [11, 14, 15]. The mortality was higher in the Gp210 antibody (+) group (RR = 2.38, 95% CI: 1.62–3.51). There was no heterogeneity (*I*^2^ = 0%) (Figure 5). Analysis of sensitivity evaluated the robustness of the effect (0.86, 95% CI: 0.47–1.25), and the range for all articles was 0.3–1.85 (Figure 3(c)). Begg's test showed publication bias in these 3 studies, although it was not significant (Pr > ∣*z*∣ = 0.296, continuity corrected) (Figure 4(c)).

### 3.5. Serum Levels of ALT, ALP, TBIL, and IgM in the Gp210 Antibody (+) and Gp210 Antibody (−) Groups

**Figure 6:**
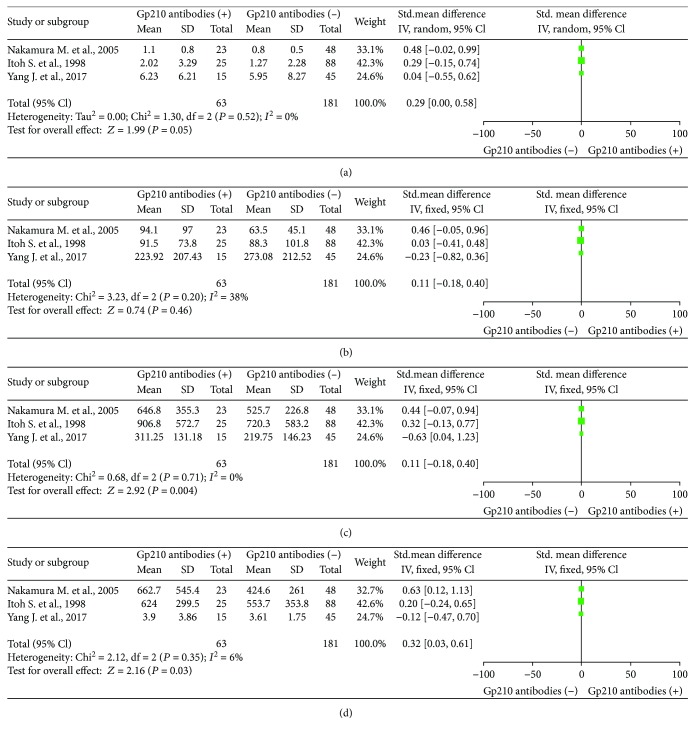
Meta-analysis of relationship between Gp210 antibody-positive rate and liver function indicators. (a) TBIL; (b) ALT; (c) ALP; (d) IgM.

The serum levels of liver function and immune indicators, including TBIL, ALT, ALP, and IgM, were measured in the Gp210 antibody (+) and Gp210 antibody (−) groups [14, 16, 17]. For liver function indicators, there was no significant difference in the serum level of TBIL between the groups (SMD = 0.29, 95% CI 0–0.58), and there was no heterogeneity (*I*^2^ = 0%) (Figure 6(a)). Similarly, there was no significant difference in the serum level of ALT between the groups (SMD = 0.11, 95% CI: −0.18 to 0.40). There was moderate heterogeneity (*I*^2^ = 38%) (Figure 6(b)). The serum level of ALP was higher in the Gp210 antibody (+) group (SMD = 0.43, 95% CI: 0.14–0.72). There was no significant heterogeneity (*I*^2^ = 0%) (Figure 6(c)). For immune indicators, there was a significant difference in the serum level of IgM (SMD = 0.32, 95% CI: 0.03–0.61) (Figure 6(d)) between the two groups.

### 3.6. Age and Number of Female Patients in the Gp210 Antibody (+) and Gp210 Antibody (−) Groups

**Figure 7:**
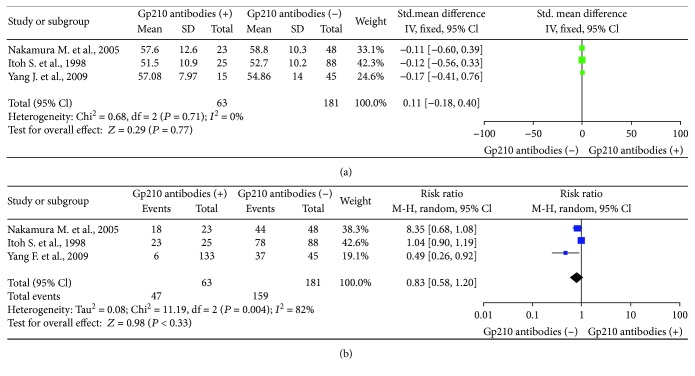
Meta-analysis of relationship between Gp210 antibody-positive rate and age or sex. (a) age; (b) sex.

The age and number of female patients were measured in the Gp210 antibody (+) and Gp210 antibody (−) groups [14, 16, 17]. There was no significant difference in age between the groups (SMD = −0.04, 95% CI: −0.33 to 0.25), and there was no heterogeneity (*I*^2^ = 0%) (Figure 7(a)). Similarly, there was no significant difference in sex between the groups (RR = 0.83, 95% CI: 0.58–1.20) with high heterogeneity (*I*^2^ = 82%) (Figure 7(b)).

## 4. Discussion

This study evaluated the published literature on the positive rate of Gp210 antibodies in anticipating the poor prognosis of PBC at the time of diagnosis. The results support that the positive rate of Gp210 antibodies is positively correlated with poor prognosis and even positively correlated with the mortality rate. Furthermore, we found that the basal level of some indicators, including ALP and IgM, are higher in the Gp210 antibody (+) group. Our results provide evidence for Gp210 antibodies as an early prognostic indicator of PBC. Combination of Gp210 antibodies, ALP and IgM may be a good prognostic tool for PBC at the time of diagnosis in the future.

**Figure 8:**
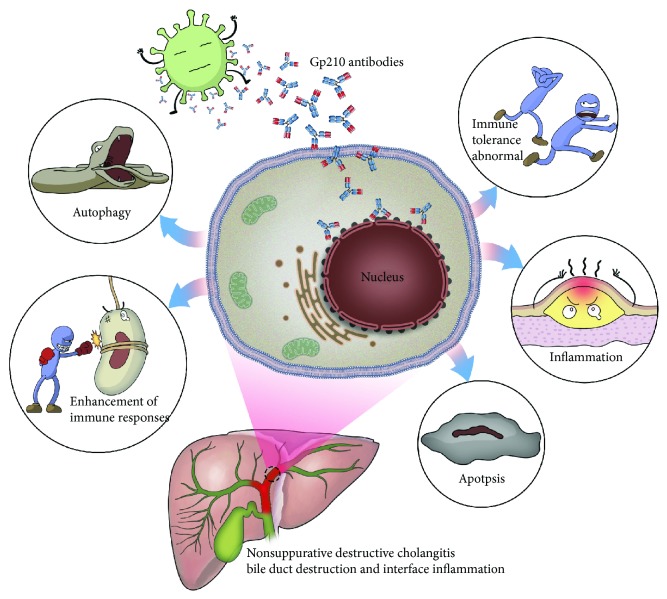
Roles of Gp210 antibodies in PBC.

Gp210 antibodies have been reported as highly specific for PBC. The roles of Gp210 antibodies in PBC are as follows. Bacterial components and other environmental triggers may be involved in the pathogenesis of PBC. These triggers, for example, bacterial lipoteichoic acid and histone-like DNA-binding protein, are detectable by synthetic Gp210 antibodies. These Gp210 antibodies, whose target antigen is a 210 kDa transmembrane glycoprotein located on the nuclear pore complex, act against an approximately 210 kDa polypeptide of the nuclear envelope. Gp210 antibodies promote apoptosis and autoantigen diffusion, break down immunological tolerance, and trigger PBC-like cholangitis, multifocal epithelial inflammation, and autoantibody production. Gp120 antibodies also sequentially upregulate innate and acquired immune responses, accompanied by autophagy and trigger nonsuppurative destructive cholangitis (Figure 8). It is widely known that the expression of Gp210 antibodies is increased on the nuclear envelope of biliary epithelial cells in small bile ducts in almost all specimens from PBC but is weak in autoimmune hepatitis and other autoimmune diseases. The level of Gp210 antibodies is positively correlated with portal inflammation, interface hepatitis, and lobular inflammation in PBC [16, 18–23].

In addition to widespread acknowledgement of their role in PBC diagnosis [24], many researchers have explored the important role of Gp210 antibodies in PBC prognosis in recent years. Nakamura et al. indicated that the increased expression of gp210 in small bile ducts, which is probably associated with inflammatory damage, is possibly involved in autoimmune response to gp210, leading to progression to end-stage hepatic failure in PBC [21]. At present, however, a large multicenter study is needed to confirm the prognostic utility of Gp210 antibodies. Our meta-analysis supported the idea that Gp210 antibodies at diagnosis are closely related to poor prognosis of PBC. Besides poor outcome, PBC has many types of progression, such as portal hypertension, liver failure, and jaundice. There are reports that liver failure in PBC is characterized by the presence of Gp210 antibodies, but other types of progression may not be so [25]. All types of PBC progression had a higher incidence in the Gp210 antibody (+) group. Compared with other types of progression, the incidence of liver failure was significantly correlated with Gp210 antibodies. The mortality in the Gp210 antibody (+) group was also significantly higher than that in the Gp210 antibody (−) group.

The serum levels of liver function indicators (such as aminotransferase, albumin, and TBIL), age, and sex are recognized as important predictors of survival in PBC after UDCA treatment [26, 27]. One study has demonstrated that the serum level of immunoglobulin can estimate a more precise probability of survival for any given patient at any time during the course of the disease [28]. Whether these indicators can predict the prognosis of PBC with Gp210 antibodies at the time of PBC diagnosis has not yet been studied. In the present study, we found that serum levels of ALP and IgM were associated with Gp210 antibodies, while serum levels of ALT and TBIL were not. Sex and age were also not associated with Gp210 antibodies. Therefore, higher levels of ALP and IgM at diagnosis are two other predictors for poor prognosis of PBC. These indicators and Gp210 antibodies can be used in predicting the prognosis of PBC at the time of diagnosis. This provides a good basis for further PBC management.

There were some limitations to our study. First, there were only 5 studies that mentioned the relationship between Gp210 antibodies and PBC prognosis, and most of them had small samples. Second, some liver function indicators were not detected in these studies, such as *γ*-glutamyl transpeptadase. Third, the patients included in this study were all Asians. However, with the development of technology, new assay methods can enhance the detection of Gp210 antibodies. More high-quality studies are required to further analyze the effects of Gp210 antibodies in the prognosis of PBC.

## 5. Conclusion

PBC-specific Gp120 antibodies are optimal predictors of PBC prognosis at the time of diagnosis. Some other liver function indicators, such as ALP and IgM, can be used as predictors to complement Gp210 antibodies to establish a stratification tool to predict the prognosis of PBC at the time of diagnosis.

## Figures and Tables

**Table 1:** The main characteristics of the included studies.

Author and year	Age	Female (Gp210 antibodies (+)/Gp210 antibodies (-))	Numbers of patients (Gp210 antibodies (+)/Gp210 antibodies (-))	Numbers of patients suffered from poor prognosis or death (Gp210 antibodies (+)/Gp210 antibodies (-))	Level of ALT (U/L), ALP (U/L), TBIL (mg/dl), and IgM (mg/dl) (Gp210 antibodies (+)/Gp210 antibodies (-))	Treatment period (months) (Gp210 antibodies (+)/ Gp210 antibodies (-))	Race
Itoh S. et al., 1998 [14]	51.5 ± 10.9 vs. 52.7 ± 10.2	12/101	25/88	Other types of poor prognosis: 11/16 Liver failure type: 8/8 Died: 10/12	ALT: 91.5 ± 73.8/88.3 ± 101.8 ALP: 906.8 ± 572.7/720.3 ± 583.2 TBIL: 2.02 ± 3.29/1.27 ± 2.28 IgM: 624 ± 299.5/553.7 ± 353.8	80.9 ± 52.9/80.9 ± 52.9	Japan
Nakamura M. et al., 2005 [15]	57.6 ± 12.6 vs. 58.8 ± 10.3	9/63	23/48	Liver failure type: 6/2 Died: 4/1	ALT: 94.1 ± 97/63.5 ± 45.1 ALP: 646.8 ± 355.3/525.7 ± 226.8 TBIL: 1.1 ± 0.8/0.8 ± 0.5 IgM: 662.7 ± 545.4/424.6 ± 261	83.7 ± 60.5/65.5 ± 50.1	Japan
Nakamura M. et al., 2007 [16]	57.5 ± 9.3	20/197	43/174	Other types of poor prognosis: 6/10 Liver failure type: 6/1	Na	75.9 ± 59.9/75.9 ± 59.9	Japan
Yang J. et al., 2009 [17]	57.08 ± 7.97 vs. 54.86 ± 14	17/43	15/45	Na	ALT: 223.92 ± 207.43/273.08 ± 212.52 ALP: 311.25 ± 131.18/219.75 ± 146.23 TBIL: 6.23 ± 6.21/5.95 ± 8.27 IgM: 3.9 ± 3.86/3.61 ± 1.75	Na	China
Yang F. et al., 2017 [11]	50 ± 10	23/253	133/143	Other types of poor prognosis: 26/11 Died: 40/21	Na	36 ± 16/36 ± 16	China

**Table 2:** The US Agency for Healthcare Research and Quality checklist for quality assessment of one-arm research.

Author and year	Is the case definition adequate?	Representativeness of the cases?	Selection of controls	Definition of controls	Comparability of cases and controls on the basis of the design or analysis	Ascertainment of exposure	Same method of ascertainment for cases and controls	Nonresponse rate
Itoh S. et al., 1998	Yes	Yes	Hospital controls	No description	Yes	No blind status	Yes	No description
Nakamura M. et al., 2005	Yes	Yes	Hospital controls	No description	Yes	No blind status	Yes	No description
Nakamura M. et al., 2007	Yes	Yes	Hospital controls	No description	Yes	No blind status	Yes	No description
Yang J. et al., 2009	Yes	Yes	Hospital controls	No description	Yes	No blind status	Yes	No description
Yang F. et al., 2017	Yes	Yes	Hospital controls	No description	Yes	No blind status	Yes	No description
